# Determination of the Effects of Transcutaneous Auricular Vagus Nerve Stimulation on the Heart Rate Variability Using a Machine Learning Pipeline

**DOI:** 10.1089/bioe.2021.0033

**Published:** 2022-09-08

**Authors:** Anna Tarasenko, Stefano Guazzotti, Thomas Minot, Mikheil Oganesyan, Nickolai Vysokov

**Affiliations:** ^1^ BrainPatch Ltd., London, United Kingdom.; ^2^ School of Physics and CRANN Institute, Trinity College Dublin, Dublin, Ireland.

**Keywords:** tVNS, taVNS, HRV, AI, ML, stress

## Abstract

**Background:**

We are all aware of day-to-day healthy stress, but, when sustained for long periods, stress is believed to lead to serious physical and mental health issues.

**Materials and Methods:**

In this study, we investigated the potential effects of transcutaneous auricular vagus nerve stimulation (taVNS) on stress processing as reflected in the electrocardiogram (ECG)-derived biomarkers of stress adaptability. Stress reflecting biomarkers included a range of heart rate variability metrics: standard deviation of N-N intervals (SDNN), root mean squared of successive differences in heartbeat intervals (RMSSD), low-frequency component, high-frequency component and their ratio (LF, HF, and LF/HF).

In addition, we created a machine learning model capable of distinguishing between the stimulated and nonstimulated conditions from the ECG-derive data from various subjects and states. The model consisted of a deep convolutional neural network, which was trained on R-R interval (RRI) data extracted from ECG and time traces of LF, HF, LF/HF, SDNN, and RMSSD.

**Results:**

Only LF/HF ratio demonstrated a statistically significant change in response to stimulation. Although the LF/HF ratio is expected to increase during exposure to stress, we have observed that stimulation during exposure to stress counteracts this increase or even reduces the LF/HF ratio. This could be an indication that the vagus nerve stimulation decreases the sympathetic activation during stress inducement.

Our Machine Learning model achieved an accuracy of 70% with no significant variations across the three states (baseline, stress, and recovery). However, training an analogous neural network to identify the states (baseline, stress, and recovery) proved to be unsuccessful.

**Conclusion:**

Overall, in this study, we showed further evidence of the beneficial effect of taVNS on stress processing. Importantly we have also demonstrated the promising potential of ECG metrics as a biomarker for the development of closed-loop stimulation systems.

## Introduction

We are all aware of day-to-day healthy stress, but, when sustained for long periods, stress is believed to lead to serious physiological and mental health issues.^1–3^ At the time of writing this article, the global pandemic and the changes imposed by related restrictions are having a profound effect on the mental health and well-being of people around the world.^4^ This created an unprecedented demand to develop effective tools for measuring and controlling stress to prevent stress-related disorders and disabilities. This is a complex task requiring a multifaceted approach and in this article, we investigated the potential for noninvasive brain stimulation and heart rate-derived parameters for this purpose. However, to recapitulate stress in a well-controlled environment, we had to limit the definition of stress to one that is specific to our research as a “lasting unpleasant emotional sensation triggered by an external visual cue.”

When looking at heart rate-derived parameters, one of the key features of a healthy heart is the ability to quickly react to various stressors. This property is controlled by a network of complex mechanisms and can be jointly referred to as heart rate variability (HRV). Various measures were proposed to both quantify and analyze HRV.^5^ These include time-domain analysis techniques (e.g., standard deviation of N-N intervals [SDNN], root mean squared of successive differences in heartbeat intervals [RMSSD]), and frequency domain techniques of analyzing HRV band powers and their ratios (e.g., high frequency [HF], low frequency [LF], and LF/HF ratio).

HRV is obtained by calculating the time intervals between the heartbeats—RR intervals. It is believed that inter-beat intervals reflect the balance between the sympathetic and parasympathetic nervous systems. Therefore, the aforementioned measures are associated with the activation or deactivation in either sympathetic or parasympathetic nervous systems.^5–7^ Studies investigating the effects of stress on HRV have shown that it results in a decrease in the RR intervals, increase in LF/HF ratio, mixed results in changes to SDNN, consistent decrease in RMSSD.^8–10^ Therefore, potentially these can be used as biomarkers for stress.^10–12^

However, there is a high degree of contradiction in research with regard to the direction of change of these parameters and their relative importance for stress discrimination.^13^ Therefore, for the purposes of this study, we did not limit ourselves to one particular metric and included all of them in the analysis pipeline. In the literature, there is a small number of studies that used these features to design machine learning (ML) models, which would identify stressful and resting states.^14,15^ Nevertheless, there is a lack of studies that look at the change in HRV factors before, during, and after stress inducement within the same sample. As a result, we have a poor understanding of the dynamics of HRV throughout stressful situations.

One of the key structures responsible for the activity of the parasympathetic nervous system is the vagus nerve. Because of its extensive network of innervated internal organs, it is able to affect vascular, digestive, and immune activities.^16–18^ The functional magnetic resonance imaging studies of vagus nerve stimulation (VNS) demonstrate a decrease in activation of the limbic system after the stimulation.^19,20^ This is important because the limbic system is often hyperactive in people with psychiatric disorders such as major depressive disorder and general anxiety disorder.^21^ It has been suggested that the vagus nerve is able to affect hypothalamic-pituitary-adrenal (HPA) axis, which is responsible for stress response and is affected in the cases of emotional dysregulation.^22^

At present, invasive VNS has been Food and Drug Administration (FDA) approved as a treatment for refractory major depression and intractable epilepsy.^23^ Moreover, the noninvasive analogue was widely investigated as to whether it can bring similar benefits without surgical risks. There are data to suggest positive effects in depression, anxiety, irritable bowel syndrome, rheumatoid arthritis, tinnitus, and Crohn's disease.^16,18,22–28^ Nevertheless, an important caveat is that although it might seem that there is a positive relationship between the degree of vagal activation and well-being, this notion has been challenged. There is evidence to suggest a nonlinear relationship between the vagal tone and well-being.^29^ This further demonstrates the need for a personalized system of stimulation rather than generic protocols. Our study is one step toward the development of such a system. Therefore, the aims of our study were as follows:

- To investigate potential effects of transcutaneous auricular vagus nerve stimulation (taVNS) on stress processing as reflected in the biomarkers of stress adaptability calculated from electrocardiogram (ECG).
- To create an ML model capable of distinguishing between the stimulated and nonstimulated conditions from the ECG data, and generalized to various subjects and states.

## Materials and Methods

**FIG. 1.**
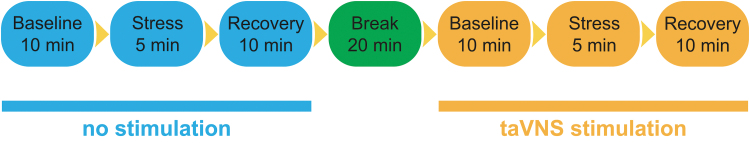
Experimental design. There were two sessions: no stimulation and stimulation. Each lasted 25 min and contained 10 min of baseline, 5 min of stress inducement, and 10 min of recovery. There was also a 20 min break in between the sessions.

Details of materials and methods, including information on ML algorithms are in the Supplementary Materials. In brief, within-subject design was used; ECG was recorded from healthy volunteers during a stressful video paradigm with and without taVNS (Fig. 1). Experimental design broadly adhered to the recommendations put forward in the literature.^30^ All the participants were debriefed about the experimental aims and procedures; informed consent was obtained before the beginning of the experiment. The design of the experiment and all associated procedures adhered to the WMA Declaration of Helsinki. The ECG data were extracted and analyzed using conventional methods as well as using deep learning. The results are presented in the following section.

## Results

### Statistical analysis of the effect of stress on HRV

**FIG. 2.**
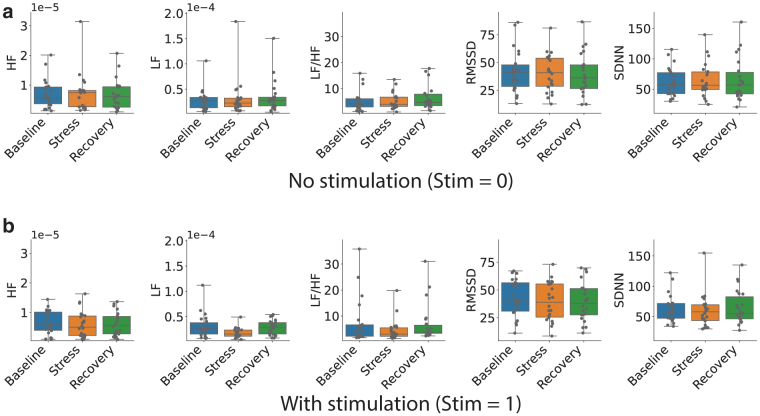
Distribution of metric values derived from ECG as described in section S1.6 in Supplementary Material across subjects for each *state* for both conditions: with **(b)** and without **(a)** stimulation. The values do not have units for the frequency domain metrics (LF, HF, and LF/HF), and they represent spectral power for the respective bands. For the time domain metrics (RMSSD and SDNN), the values are in milliseconds. The box and whiskers refer to distribution parameters as described in section S1.7 in Supplementary Material. ECG, electrocardiogram; HF, high frequency; LF, low frequency; RMSSD, root mean squared of successive differences in heartbeat intervals; SDNN, standard deviation of N-N intervals.

Before establishing the effects of the stimulation on stress management, it was important to test whether the experimental design could induce stress in the participants and which of the measured ECG-derived parameters were affected by the stress inducement. To accomplish this, we selected the data from the nonstimulated section of the experiments, Stim = 0, and tested whether stress inducement affected each parameter individually. The distribution of values across the different metrics and stress states are shown in Figure 2a.

**FIG. 3.**
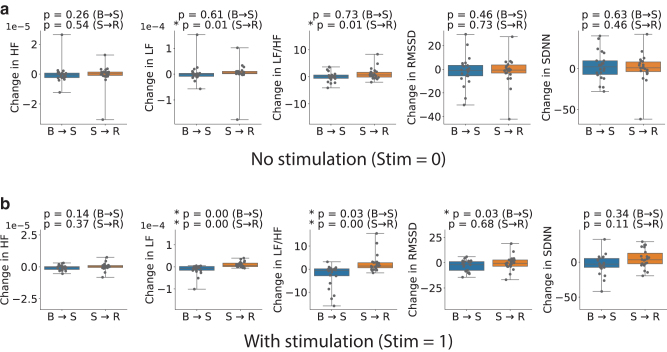
Distribution of the *changes* in values of ECG-derived metrics between *neighboring states*, that is, baseline to stress (B→S) and stress to recovery (S→R) for both conditions: with **(b)** and without **(a)** stimulation. The values do not have units for the frequency domain metrics (LF, HF, and LF/HF), and they represent spectral power for the respective bands. For the time domain metrics (RMSSD and SDNN), the values are in milliseconds. We quantify the statistical significance of the change by testing against the null hypothesis that the population mean of the changes in metric is equal to 0 through the Wilcoxon signed-rank test. The resulting *p*-values are listed in the title of each panel.

The presence of a difference between the three subsequent states was quantified by using a Wilcoxon signed-rank test. It looked at the difference in value between the selected metric for each of the two combinations of states for each subject and tested whether the two samples come from populations with the same distribution. The *p*-values obtained from the tests are listed in the title of each panel of Figure 3a for comparing the distributions of baseline to stress (B → S) and stress to recovery (S → R) metrics. The panels themselves show the distributions of these differences and highlight the position of their quartiles. The same process was then repeated for the data collected during stimulation, leading to Figures 2b and 3b.

There are trends that are commonly reported in the literature after stress inducement, which we were expecting to see in our data. These include increase in LF/HF ratio after stress inducement and decrease in RMSSD.^8–12^

As can be seen from Figure 3a and b, the observed trends were somewhat different. Figure 2a and b shows that there was a lot of interindividual variability in all the metrics. In the nonstimulated condition (Fig. 3a), there was no statistically significant difference between the baseline and stress in any metric. There was a statistically significant change in LF and LF/HF in the transition between stress and recovery. Both LF and LF/HF increased going from stress to recovery. This is the opposite of what we expected. It might be caused by a delay in stress processing, meaning that people did not get stressed immediately after the beginning of the video but a bit later. In the stimulation condition, the situation was similar. There was a statistically significant increase in the LF/HF ratio between the stress and recovery states. But also, there was a statistically significant decrease in the LF/HF ratio and a decrease in RMSSD between the baseline and stress states.

### Statistical analysis of the effect of taVNS on HRV

**FIG. 4.**
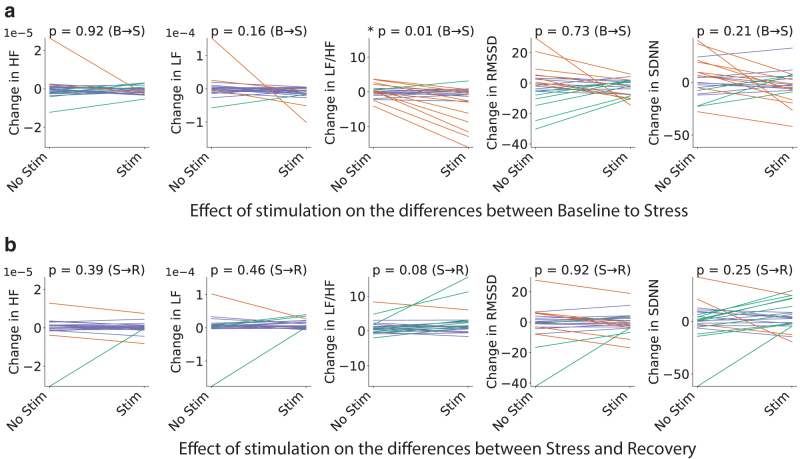
Effect of stimulation on change in HRV metrics between neighboring states, i.e. baseline to stress **(a)** and stress to recovery **(b)**. Values on the left show if a metric increased (>0) or decreased (<0) in going from one state to the next when no stimulation is applied. Values on the right correspond to the change in the value of the same metric for the stimulated condition. Lines joining the points are colored depending on whether they represent an increase (green), a decrease (orange), or a stationary value (purple). The threshold between increase, stationary, and decrease is set at 0.5×σ′, where σ′ is the standard deviation obtained from all the changes between the two relevant states with and without stimulation. *p*-Values in the title come from the Wilcoxon signed-rank test between the values on the left (Stim = 0) and on the right (Stim = 1) of each plot. HRV, heart rate variability.

Overall, 15 people out of 22 reported feeling more relaxed during stress inducement if the stimulation was applied compared with the absence of stimulation. The rest reported not feeling a difference. To quantify the effect of applying taVNS stimulation on a subject, while at the same time taking into account interindividual variability, we focused on the differences between the two subsequent states (baseline-stress and stress-recovery) with and without stimulation as derived in Figure 3a and b. For ease of comparison, we have plotted the two conditions on the same graph. Figure 4a shows the stimulation's effect on changes between baseline and stress states (B → S). Similarly, Figure 4b shows the effect of stimulation on changes that occur between stress and recovery (S → R).

Each point on the left corresponds to a change in the relevant metric for a given subject when no stimulation was applied. Corresponding change with stimulation applied is marked on the right and joined by a line to reflect on the individual effects of the stimulation. For visualization purposes, the color of each line is determined by the difference in metric changes between left (Stim = 0) and right (Stim = 1) of the plot where orange marks a clear decrease, green a clear increase, and purple a negligible effect, that is, a change smaller than half the standard deviation of all the metric changes between the two relevant stress phases with and without stimulation. As before, the statistical significance of the change was evaluated through the Wilcoxon signed-rank test of the points on the left and right of each panel, leading to the *p*-values indicated in the title.

In the literature, there is a proposed concept of responders and nonresponders to stimulation.^26^ Moreover, it seems that the presence or absence of the response might be related to the baseline LF/HF value. In the LF/HF section of Figure 4a there are two similar-sized groups of subjects whose LF/HF decreases or stays stationary. The former might be considered responders to stimulation given the presence of an effect on the HRV metric. Similarly, the latter could be identified as nonresponders, given the absence of the response. The Pearson correlation coefficient between stimulation effect and baseline (nonstimulated) value was evaluated to test whether the baseline value can predict the association with a particular category. The results show that there is a weak but statistically significant correlation (p<0.05) in the LF/HF metric between the stimulation effect and the baseline value for both the baseline to stress (r=−0.64,p=0.001) and stress to recovery transitions (r=0.51,p=0.015).

The negative correlation between the baseline value and the stimulation effect in the stress condition means the higher values of LF/HF in the baseline predict the bigger decrease in the LF/HF ratio in the stress state. Thus, implying that people with the higher baseline LF/HF ratio are more susceptible to the effects of stimulation and can be classified as responders. This is supported by the literature.^26,31^ In turn, the positive correlation of r=0.51 between the baseline LF/HF ratio and the effect of stimulation between the stress and recovery means that the higher the baseline, the higher is the stimulation-induced increase in LF/HF from stress to recovery.

### ML: discerning stimulated and nonstimulated conditions

**FIG. 5.**
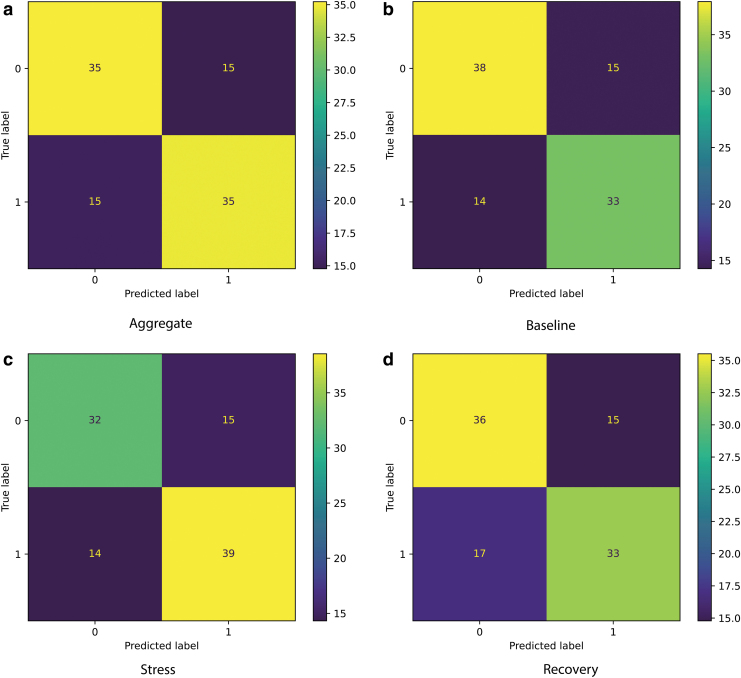
Confusion matrices for discerning stimulated (Stim = 1) and nonstimulated (Stim = 0) conditions. The numbers inside the panels represent percentages of the total number of samples (1760 from 22 individuals) in the test set. Panel **(a)** is an aggregate across all three states, **(b)** baseline, **(c)** stress, and **(d)** recovery. Here the matrices **(b, c, and d)** are a decomposition of matrix **(a)**.

By employing a convolutional neural network trained on the HRV data to discern stimulated versus nonstimulated conditions, we obtained a performance of 70% for both conditions and an F1 score of 69%. As was described in the section S1.8 in Supplementary Material, the two categories of data were fed into the model: temporal RRI series and their transformations in the frequency space (LF, HF, their ratio, RMSSD, and SDNN). Feeding both to the model yields better performance than training separately (data not shown). Figure 5 shows the confusion matrices first totaling all samples and then breaking them into confusion matrices for different states (baseline, stress, and recovery). The proportion of true labels coinciding with the predicted labels is 0.38 and 0.33 for the baseline state, 0.32 and 0.39 for the stress state, and 0.36 and 0.33 for the recovery state.

Interestingly, although misclassification is similar in baseline and stress states, it is somewhat different in the recovery state. The proportion of cases classified as stimulated while coming from the nonstimulated condition is 0.15. In contrast, the proportion of cases classified as nonstimulated while coming from the stimulated condition is 0.17. This slight bias toward the non-stimulated condition shown by the ML classifier might indicate that features correlated with stimulation presence are different in the recovery state compared to baseline and stress.

**FIG. 6.**
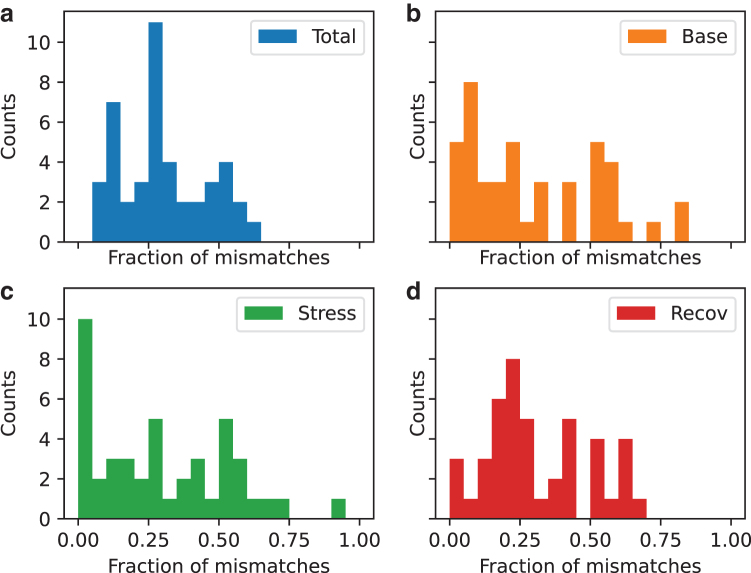
**(a)** Distribution of mismatches between the true and predicted labels in stimulation condition classification across individual subjects, including the source of these mismatches across the three different states. The percentage of mismatches was calculated for each session (2 conditions and 22 subjects) across the 40 cross-validation iterations and placed into histogram bins 5% wide. **(b–d)** A similar histogram where the data were restricted to those laying in the baseline, stress, and recovery state, respectively.

To identify whether the model performed equally well across all individuals in determining whether they were stimulated or not, we identified the number of mismatches for each individual for each condition. To visualize the results and look into the reasons for the misclassifications, we expressed them as a fraction of mismatches and plotted the distribution in Figure 6.

We can see that there is no individual with complete accuracy and only one individual with >60% mismatches. There are only around seven individuals with >50% mismatches in total. Overall, in all three states, the distribution of mismatches seems to be broadly distributed with the different degrees of the shift to the left from 50% (Fig. 6a). In particular, in the baseline state, the stimulation is more accurately determined as the distribution is shifted mainly to the left of 50%, with the tallest peak at ∼10% (Fig. 6b). Similarly, the stress condition is still left-shifted, with a tall peak at ∼5% mismatches and an even broader overall distribution (Fig. 6c). Recovery, however, is a bit worse, with the center of the distribution lying closer to 50% (Fig. 6d).

### ML: discerning baseline, stress, and recovery states

On the task of classifying the session state, that is, baseline, stress, and recovery, two experiments were performed: baseline/stress binary classification and three-way baseline/stress/recovery classification. We built the train and test set by including the samples corresponding to the states of baseline and stress for the binary classification task and the recovery state for the three-way task. This way, each state is represented equally in the test set, with each test set containing 1760 samples.

**FIG. 7.**
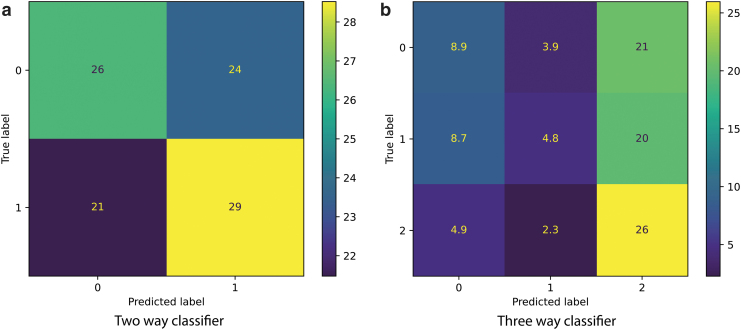
**(a)** Confusion matrix for discerning baseline and stress states in a binary classification mode across the stimulated and nonstimulated conditions. In this figure the labels are Baseline=0, Stress=1, Recovery=2. The numbers inside the panels represent percentages of the total number of samples (1760 across 22 individuals) in the test set. **(b)** Confusion matrix for discerning baseline, recovery, and stress states in a three-way classification mode across the stimulated and nonstimulated conditions.

Using this model, we achieved 55% accuracy and f1 scores for the two-way classification (Fig. 7a) and 40% accuracy, 35% f1 scores for the three-way classification (Fig. 7b).

Under the assumption that data generated by the two-way classifier can be described by the Poisson distribution, the number of correctly identified examples would be $N_{2}\approx 968\pm 31$, where 31 is the statistical uncertainty. Meanwhile, the random chance performance would add up to 880 (half of the total number of examples). This means that the obtained performance is slightly better than chance. Similarly, for the three-way classifier, the number of correctly identified examples is $N_{3}\approx 704\pm 27$ as compared with 587 obtained by chance. Hence its performance is also slightly better than chance. Interestingly, the classifier seemed to overfit the recovery state and incorrectly predict it for both stress and baseline state.

## Discussion

First, in this study, we have provided further evidence for the beneficial effects of taVNS reflected in HRV metrics. In Figure 4a we show that the stimulation is effective at reducing the expected LF/HF increase in response to stress and even reducing it below the baseline in several cases. This is important because we know from the literature that there is a disproportional increase in LF/HF in response to stress in people with affective disorders, such as anxiety.^32,33^

Our results are in line with the findings on the effects of the transcutaneous VNS (tVNS) on the cervical branch of the vagus nerve. It was shown that tVNS decreases sympathetic activation in response to stress in healthy individuals^31,34^ as well as the people suffering from post-traumatic stress disorder (PTSD).^35^ All our participants tolerated the stimulation well, with only one reporting a light muscle tension in the neck region during the stimulation, which disappeared as soon as the stimulation stopped. This goes in line with mounting evidence of the safety of tVNS in the course of prolonged stimulation periods.^36^

Second, we have created and trained an algorithm that can discern between stimulation and no stimulation conditions in various states (baseline, stress, and recovery) with a high degree of reliability (70%). Because the research in this area still remains limited, in particular in different affective states, it is difficult conclude the meaning of this accuracy value as there is no benchmark. Based on the spread of the distribution of mismatches shown in Figure 6, it might be that the features associated with stimulation are the most prominent in the baseline condition (Fig. 6b), with stress condition following closely. In the recovery state, features seem to be the least pronounced. The fact that the distributions of mismatches vary across the different states might be indicative of the fact that the stimulation effects also vary in different states. This is under the assumption that the features learned by the model are indicative of the beneficial effects of stimulation.

The state dependence of the response to the taVNS we noted in Figure 4a and in the correlation analysis has been noted before in the rat studies using invasive VNS. VNS can reduce the overarousal present in rats that are stressed at the baseline, however, has no effect on the arousal in the nonstressed rats.^37^ Vagus nerve receives numerous afferent projections that indicate the state of the viscera. If the subject is already in a calm state, with a high level of parasympathetic activity, the stimulation may not be as effective. This fits well with the polyvagal theory that focuses on the adaptive properties of the physiological state.^38^ According to this theory, the physiological state defines the range of reactions that are possible to a given stimulus. There is also an interesting hypothesis that the release of adrenalin in response to stressful situations affects the brain through the vagus nerve.^39^

The released adrenalin might bind to the beta-adrenergic receptors on the vagus nerve, which then increases the release of norepinephrine in the brain as a homeostatic mechanism to counteract stress. This was proposed as a mechanism of effectiveness of tVNS in the fear extinction in PTSD. This could potentially explain why the differences between baseline and stress states were enhanced after the stimulation. Nevertheless, this hypothesis has only been tested in animal models with invasive VNS. In addition, it is important to mention a lack of studies on continuous changes in HRV after stress inducement. These are essential to understand the effects of stimulation on the stress response, especially given the contradictory findings about the importance of HRV markers in stress identification.

The ability to discern the presence of the stimulation from the HRV data using a small section (1 min) might imply that stimulation effects can be used as optimization features for the closed-loop system. Moreover, this might also mean that this model can be further adapted for online data processing to adjust the stimulation parameters immediately. The degree of individual calibration of the model for every new user requires further investigation. We envision the need for the collection of preliminary ECG data in a range of different stress scenarios to generate a versatile model. However, going beyond 1 min for the online processing will impose a further constraint on window length for the fast Fourier transform, as that determines how far the metrics lag behind real time.

In this study, we have used simple previously reported^26^ stimulation parameters. However, optimal and desirable stimulation may require more complex biphasic patterns that would be the scope of our further investigations.

Another important part of the system that needs to be developed in parallel is the state recognition system. However, we found that both state classifiers (two-way and a three-way) are significantly worse than the stimulation condition classification model. From Figure 7a it seems the data set contains some features that allow distinguishing between the baseline and stress (and recovery) states; however, these are not prominent enough for the ML algorithm to reach a good accuracy. This leads us to believe that higher performance could be achieved by improving the quality and diversity of the collected data.

Alternatively, it might be due to the experimental design flaws, which meant that the induced stress was not sufficient to be discerned from the HRV metrics. Another possibility is that the participants were already stressed at baseline as they were expecting to see the stressful video. Using a nonstressful video as the baseline instead of the black screen might allow a clearer distinction. This, however, requires a more systematic study. Alternative stress-inducing protocols may give the model more information about the person's reaction to stress, thus enabling better recognition.

In principle, a state recognition system should conduct continuous labeling of states, rather than simple baseline-stress-recovery. To achieve this, one would need to reframe the model into a regression rather than classification. Therefore, the outcome would be a *probability* of belonging to different states. This would put the effects of the stimulation into the context of the state of a person and, therefore, enable one to bring a user closer to the desired state through the use of stimulation. However, the usefulness of HRV metrics for such purposes remains an open question given the poor performance of state classifier.

Thus, interpretation of our results has to be taken with a range of considerations that directly impact the generalizability and validity of conclusions that could be drawn.

### Experimental design considerations

There are a wide variety of stress inducement protocols used in research. However, their common drawback is the lack of realistic scenarios. Common stress paradigms include cold water immersion, needle prickling, and electric shock.^40,41^ The advantage of these protocols is that they are more generic and less dependent on individual factors such as personality and background. Nevertheless, apart from the obvious ethical implications, these paradigms are difficult to administer remotely. Therefore, in the context of this study, alternatives were used.

Although the stressful video paradigm used was easy to administer remotely, it is similarly not representative of the real-life stressors most people face. Further development would be to explore other modalities and sensory inputs for inducing specific mental states, especially combining them to produce a more immersive environment (videos, auditory and haptic inputs, and virtual reality). Our experimental paradigm was aiming to recreate the scenario of acute stress and demonstrate the change in the response to it. The effects of tVNS in the conditions of chronic stress require further investigation and more complex experimental designs.

Another important consideration is the duration of stress inducement: it was twice shorter than the baseline and recovery, thereby providing much less information for the training of the ML model. This potentially might contribute to the poor performance on the state recognition task. This is further supported by the fact that the classifier seemed to overfit the recovery state slightly (Fig. 7b).

Finally, in our design we were comparing response to a stressful video without and with stimulation within the same individuals to reduce interindividual variability assuming that any placebo effects of the stimulation were relatively small. This was done adhering to the recommendation on conduction of the HRV-based studies.^30^ A double-blinded placebo controlled study involving a sham stimulation could help further confirm the effects of the stimulation. However, due to controversies on an appropriate taVNS sham, and complexities in study design, conducting placebo-controlled study was beyond the scope of this research.

### Choice of outcome measures

According to von Rosenberg et al.,^12^ the LF/HF ratio, which is one of the key metrics in our model, does not perform equally well in different stress scenarios. Their proposed alternative is to look at the pair of values LF, HF, claiming that different combinations of high or low values of the two metrics are instrumental in identifying different kinds of stress. Yet, other research suggests that LF is a poor marker of sympathetic activity as it is influenced by the activity of both sympathetic and parasympathetic systems.^42^ Hence it might be difficult to interpret.

In addition to the HRV metrics that we used, several new “nonlinear” metrics inspired by the behavior of other nonlinear systems have been proposed, such as the sample entropy and correlation dimension.^5,43^ Even though these metrics can be, at least partially, correlated with some of the other time- or frequency-domain metrics, they could still prove useful in our pipeline as they would lead to expanded feature space for the ML analysis. Furthermore, respiration rates could be measured to control for the contribution of Respiratory Sinus Arrhythmia to HF. Finally, other ways of measuring stress, including galvanic skin response, cortisol levels, and questionnaires, could be added into the model.

### Data analysis considerations

Spline interpolation of RRI that we used, as described in Section S1.6 in Supplementary Material, does not ensure a monotonic behavior between sampling points and could, therefore, introduce artefacts in which the interpolant overshoots the sampled values, thus introducing high-frequency components. This is prevented when using more sophisticated interpolation algorithms that ensure monotonic behavior, such as cubic interpolation.^44^

The architecture used for ML training was chosen beforehand and it is possible it may benefit from the hyperpameter fine-tuning. However, this would require a separate validation set as well as a test set.

Since we performed all our analysis over a time window, some of the points in the time traces of different HRV metrics contained information from neighboring states. These can be referred to as “transition periods” between the states. If the fraction of these “transition periods” is significant, the change in the metric's value between different states could be reduced, making it harder to detect. Furthermore, having a better synchronization between collected data and the start/end of states would greatly improve our ability to remove these “transition periods.” However, this is difficult to arrange from both experimental and biological perspectives.

## Conclusion

Our findings in this project further support the promise of using taVNS in a variety of different conditions and applications suggested by previous research. In this study, we used custom-made stimulation and recording devices together with a novel method of data analysis on an example application in acute stress. By demonstrating the feasibility of using small snippets of data for determining the presence of stimulation we are getting closer to the possibility of a closed-loop taVNS system. Creating such a system requires a significant amount of further research, technological development, and trials. However, once developed, we believe that its applications will extend beyond stress and could include medical conditions that are currently trialed with taVNS such as depression, epilepsy, irritable bowel syndrome, and rheumatoid arthritis.

## Supplementary Material

Supplemental data

Supplemental data

Supplemental data
