# Supplementary material for: Determination of the Effects of Transcutaneous Auricular Vagus Nerve Stimulation on the Heart Rate Variability Using a Machine Learning Pipeline
Source: Bioelectricity. 2022 Sep 8;4(3):168–77. doi: 10.1089/bioe.2021.0033 (PMC9508455; doi:10.1089/bioe.2021.0033)
Supplement: Supplemental data [file Supp_Data.doc]

##### BIOE-2021-0033

## Supplementary Material

## S1 Supplementary Materials and Methods

### S1.1 Subjects

40 healthy volunteers were recruited to take part in the experiment. 18 recordings were rejected from the analysis because of the low quality of the collected ECG data. In the resulting sample of 22 people with a mean age of 29.1 years and a standard deviation of 6.8, there were 13 males and 9 females. All the participants were debriefed about the experimental aims and procedures; informed consent was obtained before the beginning of the experiment. The design of the experiment and all associated procedures adhered to the WMA Declaration of Helsinki. Participants filled in a short survey about their state on the day of the experiment before and after the stimulation. This was done to document any subjective experiences and potential side effects.

### S1.2 Experimental Procedure

Due to the Covid-19 restrictions, some of the experiments took place remotely. Therefore, the recording and stimulation equipment set-up was done by the participants themselves under the experimenter’s supervision via a video call. The experimenter was present throughout the whole duration of the experiment. All experiments were carried out at the same time of the day (between 11 am and 3 pm) to minimize the effect of diurnal variation. The experiment consisted of two parts, each 25 minutes long with a 20-minute break in between. Each part had 10 minutes of baseline, 5 minutes of stress inducement, and 10 minutes of recovery as shown in Fig. 1. Participants were seated comfortably on the chair with their backs resting on the back of the chair to prevent postural engagement muscles.

During the baseline and recovery, participants were asked to relax and were presented with an empty black screen to look at. For the stress inducement, the trauma film paradigm was used ^41^. Before the next session with the stimulation condition, participants were allowed to take a 20-minute break.

### S1.3 Stress Inducement

We have decided to use a trauma film, also known as the stressful video paradigm, as a means of stress inducement^41^. In particular, we picked 5-minute long footage of a post-World War II documentary (https://www.youtube.com/watch?v=CyvjcBUd-Co) from a concentration camp, henceforth referred to as the stress-inducing video. There were two different excerpts taken from the same movie. The order in which the videos were shown to participants was varied. This was because the videos could potentially be different in the degree of their emotional valence, and hence through randomly changing the order, we accounted for this. All participants were informed about the video content before the beginning of the experiment and were given the right to withdraw. The recovery period was the same as the baseline. We refer to “baseline”, “stress” and “recovery” periods as states throughout the paper.

### S1.4 taVNS Stimulation

The Transcutaneous Auricular Vagus Nerve stimulation (taVNS) was delivered through a custom made constant current stimulator. The device interface consisted of an ear-clip made of conductive rubber covered in conductive paste and was placed on the tragus of the left ear, as shown in Fig.S1. The anode was placed on the inside of the tragus. The above setup is believed to stimulate the auricular branch of the vagus nerve. The stimulation was delivered as 200 us square monopolar constant current pulses at 30 Hz throughout the entire duration of the second condition of the experimental procedure. The amplitude of stimulation was adjusted to suit individual comfort levels and ranged from 1 mA to 3.5 mA such that it was just under the sensitivity threshold. The peak voltage was monitored throughout the experiment and was no greater than 32 V. The stimulation was delivered for 25 minutes for the entire duration of the second part of the experiment. This was done to prevent contamination of the baseline by any potential lingering effects of the stimulation. No additional sham stimulation was performed as explained in section 3. Within the paper the term “condition” refers to the stimulation status, i.e. whether it was on (Stim = 1) or off (Stim = 0).

### S1.5 ECG Recording

ECG recording was done with a standard three-lead placement and OpenBCI Cyton equipment and user interface. The cathode was placed under the right clavicle, anode under the left clavicle and the ground under the left rib ^45^. The sampling frequency of the equipment was 250 Hz.

### S1.6 ECG Processing

Once the ECG has been collected, we performed the first step of processing using the *NeuroKit* package^46^. We removed any long term trends/drifts and overall DC component as well as filtered out any spurious frequency as described in Makowski et al.^46^. We again used *NeuroKit* to identify the occurrence of R peaks, remove artefacts, as described in Lipponen and Tarvainen^47^, leading to a sequence of times at which the R peaks occur. We then calculated the time interval between adjacent R peaks to get what is commonly known as an RR interval (RRI), which acted as a foundation input for the ML model.

Following the findings of previous studies, we also calculated some of the metrics commonly extracted from ECG ^5–7^. The SDNN and RMSSD were directly calculated from the RRI trace in a window of length *W* = 5 minutes which was moved along the time series with a step of 5 s. To calculate the frequency domain metrics, we first performed a *spline* interpolation of the RRI traces to achieve a constant sampling of $4 \text{Hz}$. We then proceeded by applying a windowed Fourier transform to these re-sampled traces – using the same window parameters as for the time domain metrics – estimated the periodogram and finally calculated the total power in the LF ($0.04 \text{Hz}$ to $0.15 \text{Hz}$) and HF ($0.15 \text{Hz}$ to $0.40 \text{Hz}$) spectral bands. Finally, the LF/HF was calculated as the ratio of the power in the two spectral bands, leading to a total set of 5 metrics.

All the metrics time traces (LF, HF, LF/HF, SDNN and RMSSD) were averaged within the time of a specific state, i.e., *baseline*, *stress* or *recovery*. As a consequence of the windowing process, values of a metric at the border between two states could include a minority contribution from RRI points from the neighbouring state. At the end of this process, a single number was produced for each subject for each possible combination of the following parameters:

• Metric (M), which can be one of LF, HF, LF/HF, SDNN, or RMSSD;

• Stress state (T), which can be one of baseline (B), stress (S), or recovery (R);

• Stimulation condition (Stim), which can be either on (1) or off (0).

### S1.7 Statistical Analysis

The statistical analysis was conducted in Python 3.8, using statistical functions provided by the 1.6.2 version of the Scipy package. Testing the distributions of collected samples for normality revealed that most of them are not normally distributed, thereby supporting our decision to apply non-parametric statistical testing. A *p*-value of $p<0.05$ was considered statistically significant. The plots were generated using the Seaborn Python library and show the distribution of values via a colored box going from the first to the third quartile, a horizontal black line at the median and black whiskers extending to the smallest and largest values.

### S1.8 Machine Learning

Discriminating between stimulated and non-stimulated conditions was formulated as a binary classification task. Deep learning was the method of choice due to its ability to learn non-linear features and easily accept multi-modal inputs such as RRI traces and raw spectral information. More specifically, an architecture based on one dimensional (1D) convolutions was employed. Convolutional neural networks have been used with great effect in various tasks such as image recognition, and more recently, have been applied to ECG classification tasks ^48^. Therefore, the model architecture was a series of convolutions, each followed by ReLU and batch-norm layers, as shown in Fig. S2. The last stage was fully connected and outputted a soft-max classifier (2 or 3 units depending on whether the task is to classify stimulation/non-stimulation or baseline/stress/recovery). The model was trained on the cross-entropy loss with ADAM optimization. Finally, performance on the test set was measured using the accuracy and f1 scores.

As mentioned in the introduction, the aim of the experiment was to produce an ML classifier that generalises to different subjects and states. Thus, the training and validation data sets were sampled to represent all experiment participants’ sessions. The data set consists of time series with RRI and its associated traces of frequency and time transforms (i.e., LF, HF, LF/HF, RMSSD and SDNN) stacked as individual channels. For the transformed channels, the window size was set at 120 seconds, a stride at 0.5 seconds, and the frequency transforms were calculated after re-sampling the RRI to 4 Hz. For consistency with the transformed series, the first channel was set to the re-sampled version of the RRI trace with a stride of 0.5 s.

Each “sample” used for training or test was a trace of 100 points (corresponding to 50 seconds) long. Samples taken for the test set (one sample per session/condition per subject) were selected randomly. In contrast, the remaining sequences (equivalent to about 15 non-overlapping samples) in each session were then re-concatenated and used as the training set. We ensured that the test split was balanced across the stimulation conditions and the session states (baseline, stress, recovery). When training, batches (typical size of 128 or 256 samples per batch) were generated by randomly selecting the samples across the whole training set. One epoch was then defined by multiplying the lengths of the batch and input sequences and iterating passes over the generated batches until the total seen length amounted to that of the training set. For a batch size of 256, it corresponds to two passes for completing an epoch. This was repeated for 20,000 epochs to ensure convergence. Finally, the performance of the model was validated against the test set. In these experiments, the method used for updating the weights in the network is backpropagation with the ADAM optimizer, as we compute the cross-entropy loss between the prediction and ground truth of the test set. To ensure the model’s generalizability, we performed 40 cross-validation iterations, generating a new test set for each one. Thus, for example, for the stimulation condition classification, the total number of samples in the test set was 1760 (or approximately 10% of the total set of samples), as they were randomly selected for each of the 22 subjects, the two conditions, and across 40 cross-validation iterations. The overall accuracy was computed as an average across iterations. The numbers of correctly and incorrectly identified samples were aggregated across all the iterations to generate confusion matrices where the numbers represent a fraction of the total number of samples.

Figure S1: Stimulation Earclip. The custom designed earclip was 3D printed in soft polymer (TPU95). It allowed positioning and securing of the anode and cathode on the inner and outer parts of the tragus respectively for the transcutaneous stimulation of the auricular branch of the vagus nerve. The wires (red and brown on the photo) were connected to custom built current-driven stimulation device capable of delivering up to $\pm32$ V and up to 5 mA of current. The wires were connected to an inert conductive material (conductive rubber) and ample Ten20 paste was used to interface with the skin and allow safe delivery of the current.
