# Supplementary material for: Determination of the Effects of Transcutaneous Auricular Vagus Nerve Stimulation on the Heart Rate Variability Using a Machine Learning Pipeline
Source: Bioelectricity. 2022 Sep 8;4(3):168–77. doi: 10.1089/bioe.2021.0033 (PMC9508455; doi:10.1089/bioe.2021.0033)

Figure S1: Stimulation Earclip. The custom designed earclip was 3D printed in soft polymer (TPU95). It allowed positioning and securing of the anode and cathode on the inner and outer parts of the tragus respectively for the transcutaneous stimulation of the auricular branch of the vagus nerve. The wires (red and brown on the photo) were connected to custom built current-driven stimulation device capable of delivering up to $\pm32$ V and up to 5 mA of current. The wires were connected to an inert conductive material (conductive rubber) and ample Ten20 paste was used to interface with the skin and allow safe delivery of the current.


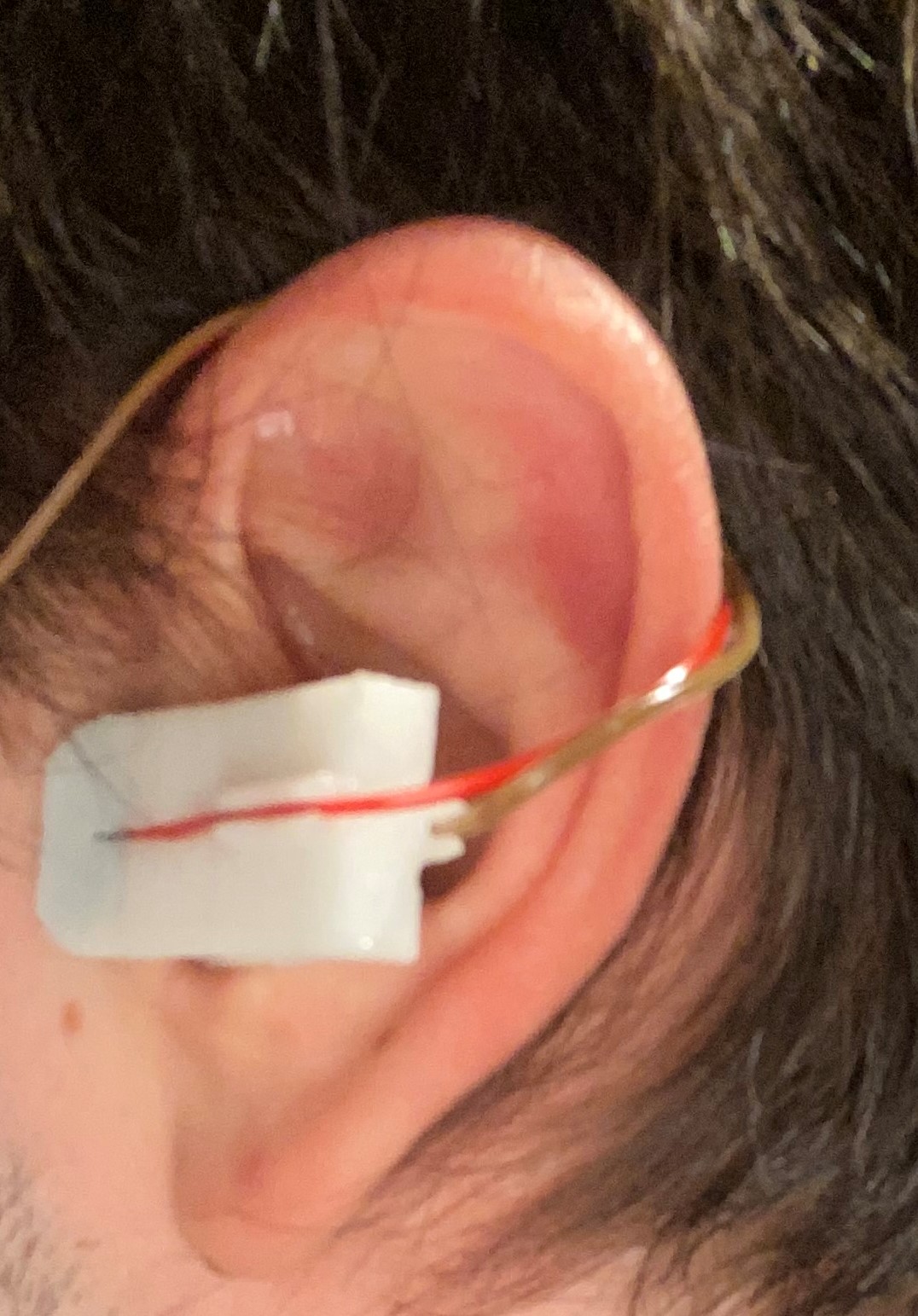

Supplement: Supplemental data [file Supp_FigS1.docx]
