# Supplementary material for: Determination of the Effects of Transcutaneous Auricular Vagus Nerve Stimulation on the Heart Rate Variability Using a Machine Learning Pipeline
Source: Bioelectricity. 2022 Sep 8;4(3):168–77. doi: 10.1089/bioe.2021.0033 (PMC9508455; doi:10.1089/bioe.2021.0033)

Figure S2: Network Architecture Design. The model has a 5-layer convolutional architecture. The first stage represents the input data where there are 5 inputs channels for the different metrics, and 1x100 are the dimensions (1D) of the convolution kernel, while the last stage is a fully connected feature vector used for the softmax binary classification output. The numbers describe the parameters of the layer, e.g. conv1 has 24 filters and the output dimensions are 1x60


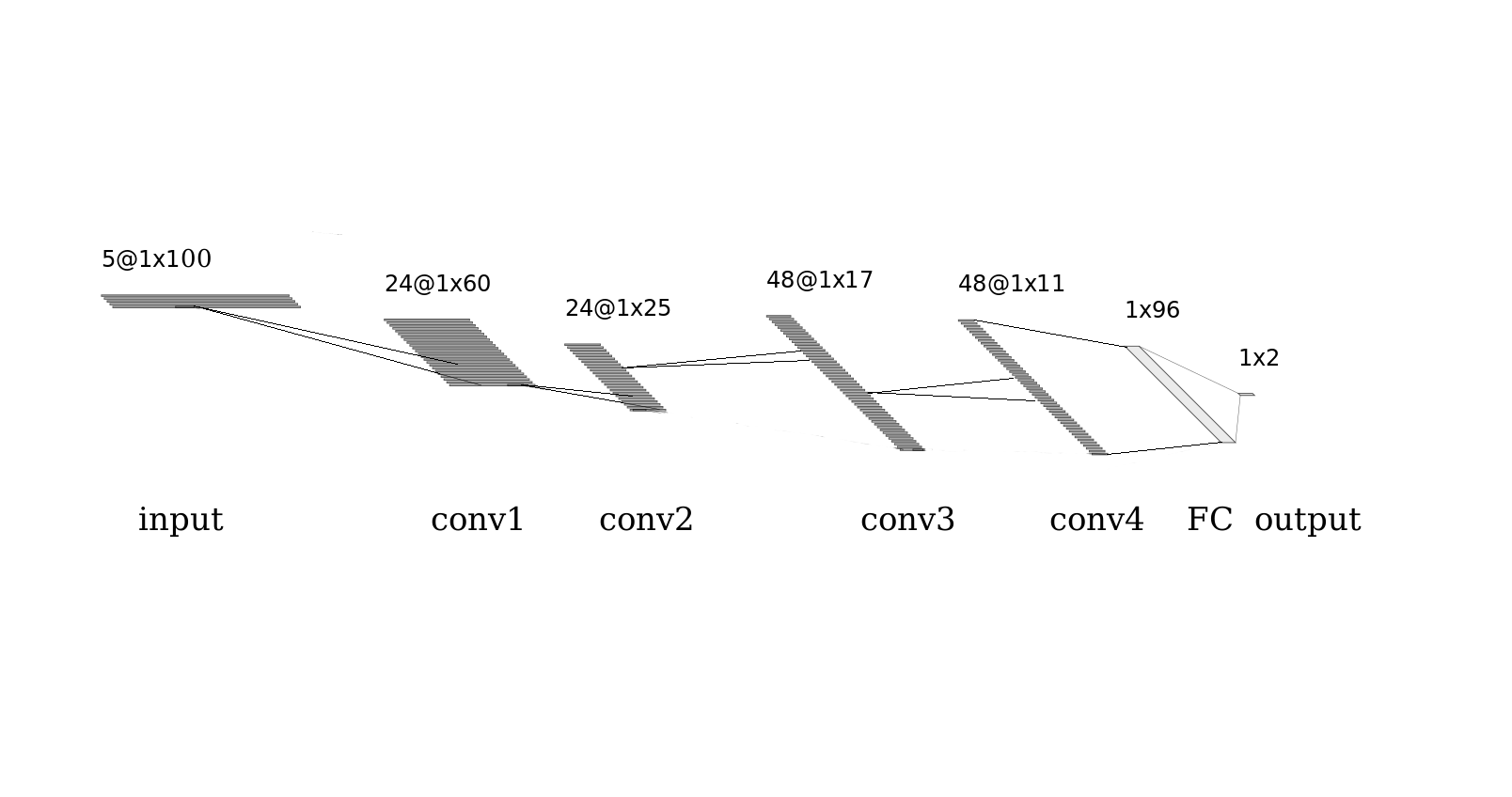

Supplement: Supplemental data [file Supp_FigS2.docx]
